# A big data analysis of Twitter data during premier league matches: do tweets contain information valuable for in-play forecasting of goals in football?

**DOI:** 10.1007/s13278-021-00842-z

**Published:** 2021-12-29

**Authors:** Fabian Wunderlich, Daniel Memmert

**Affiliations:** Institute of Exercise Training and Sport Informatics, German Sport University Cologne, Köln, Germany

**Keywords:** Big data, Data mining, Social networks, Twitter, Football forecasting, In-play forecasting

## Abstract

Data-related analysis in football increasingly benefits from Big Data approaches and machine learning methods. One relevant application of data analysis in football is forecasting, which relies on understanding and accurately modelling the process of a match. The present paper tackles two neglected facets of forecasting in football: Forecasts on the total number of goals and in-play forecasting (forecasts based on within-match information). Sentiment analysis techniques were used to extract the information reflected in almost two million tweets from more than 400 Premier League matches. By means of wordclouds and timely analysis of several tweet-based features, the Twitter communication over the full course of matches and shortly before and after goals was visualized and systematically analysed. Moreover, several forecasting models including a random forest model have been used to obtain in-play forecasts. Results suggest that in-play forecasting of goals is highly challenging, and in-play information does not improve forecasting accuracy. An additional analysis of goals from more than 30,000 matches from the main European football leagues supports the notion that the predictive value of in-play information is highly limited compared to pre-game information. This is a relevant result for coaches, match analysts and broadcasters who should not overestimate the value of in-play information. The present study also sheds light on how the perception and behaviour of Twitter users change over the course of a football match. A main result is that the sentiment of Twitter users decreases when the match progresses, which might be caused by an unjustified high expectation of football fans before the match.

## Introduction

In recent years football analysis has increasingly benefited from Big Data analysis and machine learning methods, in particular in an attempt to understand tactical behaviour and identify success-enhancing strategies (Dick and Brefeld 2019; Grunz et al. 2012; Memmert and Raabe 2018; Rein and Memmert 2016). The present paper puts the approach of Big Data analysis and machine learning into a slightly different context by incorporating Twitter data into the analysis. It focuses on in-play forecasts in football by examining the question whether information becoming available during a match is valuable to forecast the further course of events. This analysis is relevant to better understand football-related Twitter communication, to assess the role of randomness in football and valuable for coaches, match analysts and broadcasters to better understand the influence of in-play events on the further course of a match.

The forecasting literature reflects two important aspects researchers have faced when investigating predictive tasks in football. The first aspect of forecasting is statistical and related to developing team ratings and forecasting models with the best possible ability to derive forecasts from obvious predictors such as prior match results. One of the most prominent approaches is to estimate offensive and defensive strength parameters of the teams and use these as inputs for probability models including Poisson models (Koopman and Lit 2015; Maher 1982), birth process models (Dixon and Robinson 1998) and Weibull count models (Boshnakov et al. 2017). Other researchers have used regression models based on one or various covariates such as Hvattum and Arntzen (2010) using ELO ratings in combination with an ordered logit regression model or Goddard and Asimakopoulos (2004) using various covariates in an ordered probit regression model. The present approach is primarily related to the second aspect of forecasting, which is data-based and attempts to identify and investigate further sources of information that prove useful in football forecasting. One source of information obviously is betting odds (Forrest et al. 2005) being interpreted as a forecast and used as a standard benchmark. Further sources include human forecasts (Andersson et al. 2005), prediction markets (Spann and Skiera 2009), ranking systems such as the FIFA World Ranking (Lasek et al. 2013), market values (Peeters 2018) or sets with various explanatory variables including match significance, involvement in cup competitions and geographical distance between teams (Goddard and Asimakopoulos 2004).

In the literature, football forecasting is most prominently associated with forecasting the match result in terms of win, draw or loss. This seems a little one-dimensional, in the light of the wide range of events taking place during a football match. With regard to the common win/draw/loss forecast, Koopman and Lit (2019) introduced a categorization of methods, namely models indirectly based on modelling the number of goals scored by both teams, indirectly based on modelling the goal difference or modelling the result in terms of win, draw, loss directly. Forecasting the number of goals, in that sense, is not an exotic task as models falling into the first category and often being based on Poisson distributions (Karlis and Ntzoufras 2003; Koopman and Lit 2015; Maher 1982) can easily be reused for goal forecasting. Boshnakov et al. (2017) pursue this strategy by using a Weibull count model to obtain forecasts for both match result and total number of goals. Wheatcroft (2020) uses ratings based on match statistics and logistic regression to forecast the number of goals and is—to the best of our knowledge—the only paper focusing in particular on this type of forecasting. Forecasting of total goals thus can be considered a neglected aspect in the forecasting literature, presumably driven by the fact that the match results have stronger emotional and financial consequences for the fans and teams than the total number of goals.

Another research gap in football forecasting is the investigation of forecasts made during the course of a match. This comes as a surprise as so-called in-play betting has gained significant importance for bookmakers (Killick and Griffiths 2019; Lopez-Gonzalez and Griffiths 2016). Moreover, coaches, match analysts and broadcasters are highly interested in analysing matches in-play. In fact, some researchers have put thoughts to the scoring processes during the course of the match in more detail. Dixon and Robinson (1998) use a birth process model allowing scoring intensities to change during the match and depend on the score to analyse the deviations from constant scoring rates. Similarly, Heuer and Rubner (2012) use a model-free statistical analysis to investigate in which match situations scoring intensities deviate from a constant rate. Both approaches are mainly focused on understanding the process of a football match and whether certain game situations influence the scoring behaviour. None of these articles investigates in-play forecasts by calculating the effect of scoring deviations on the accuracy of in-play forecasts. To the best of our knowledge, the only paper investigating the role of in-play information in forecasting football is the recent work of Zou et al. (2020), which, however, is limited to the number of goals as only in-play information. While our paper is limited to football, contributions focused on in-play models and in relation to in-play betting odds have been investigated in other sports such as tennis (Easton and Uylangco 2010; Kovalchik and Reid 2019) and cricket (Akhtar and Scarf 2012; Asif and McHale 2016). Reasons for the little effort made so far on in-play forecasting in football might be a higher model complexity, less availability of in-play betting odds as a benchmark in comparison to pre-game betting odds, and higher effort to gather and handle in-play data.

The difficulty of in-play forecasting of goals in football might be surprising because intuitively fans, experts and commentators commonly argue that they have anticipated a goal; they’ve seen it coming or explain it as the logical consequence of the course of play. This, however, could be a biased perception and it would be quite costly to measure the collaborative human perception of a football match and the collaborative anticipation of the further progress in an experimental approach. For that reason, we make use of an existing source of (big) data: Short textual messages from the microblogging platform Twitter with regard to a certain football match, which can be considered an in-play reflection of collaborative human perception on this match. While traditional dataset and probability models remain a predominant approach in football forecasting (Boshnakov et al. 2017; Koopman and Lit 2019; Wheatcroft 2020), researchers have also started to make use of Big Data (Brown et al. 2017) and machine learning (Berrar et al. 2019; Hubáček et al. 2019) in this domain. Twitter data itself has been used in various domains of forecasting including elections (Huberty 2015; Tumasjan et al. 2010) or stock prices (Bollen et al. 2011; Zhang et al. 2011), but have been discussed very controversial and critically (Gayo-Avello 2013; Huberty 2015; Jungherr et al. 2011). While Twitter certainly provides the possibility to gather massive datasets, the process of actually extracting relevant information is challenging and attempts to use Twitter in football forecasting have reported mixed results (Brown et al. 2017; Godin et al. 2014; Schumaker et al. 2016). In economic and political situations, the theoretical mechanism is viable as Twitter may reflect the opinion of the users and both election results and stock prices are directly influenced by the perception of the public. In football, this mechanism is evidently not present as a team will not succeed in a match only because the public would like to see the team win. In forecasting goals in-play, however, the following mechanism is conceivable: The course of the match influences the perception of the fans that will share their opinion on Twitter. If the course of play is actually a predictor for upcoming goals, Twitter data might indeed have predictive value. Though not considering predictive aspects, some researchers have focused on analysis of in-play Twitter data in relation to football matches. It has been reported that fans’ sentiments reflect reactions to goals of the own or opposing team (Yu and Wang 2015), fans tend to have a higher team identification when the team is leading than when it is trailing (Fan et al. 2020) and communication on the video assistant referee (VAR) is strongly associated with negative sentiment (Kolbinger and Knopp 2020). In contrast to the present study, however, analyses were based on highly limited sample sizes of five or less matches (Fan et al. 2020; Yu and Wang 2015) or on a very specific type of event during the matches, namely the VAR (Kolbinger and Knopp 2020).

The contributions of the present approach are threefold. First, a preliminary analysis sheds light on the general difficulty of in-play forecasting. Second, the topics discussed by Twitter users as well as their perception of the match over the course of football matches and as a reaction to goals are analysed by means of sentiment analysis techniques and further non-semantic tweet characteristics. Third, the possible informative value of Twitter data when used in in-play forecasting models is investigated.

## Data and methods

### Data

For a preliminary analysis, a dataset consisting of a total of 31,912 matches from 10 seasons (07/08–16/17) in 10 major European leagues (first divisions of England, Spain, Germany, Italy, France, Portugal, Belgium, Turkey, the Netherlands and Greece) was used. Data were obtained from http://football-data.co.uk and included the following information for each match: Teams involved, date, halftime score, final score and betting odds for over-under 2.5 goals. Betting odds can be interpreted as an aggregated market forecast and reflect a very strong benchmark for forecasting models in football (Hvattum and Arntzen 2010; Štrumbelj and Šikonja 2010). Over-under bets reflect betting opportunities on the total number of goals, in this case with the possibility to bet on *two or less* or *three or more* goals. For the main analysis, data of Premier League football matches were obtained in the period from 22 February 2019 to the last game before the interruption of the league caused by the COVID-19 pandemic on 09 March 2020. Removing three matches due to missing data, this adds up to a total of 404 matches, representing a smaller, but richer dataset. Data source and information included are analogous to the previously mentioned dataset. Additionally, it includes betting odds for over-under 1.5 goals in the second half collected from http://www.oddsportal.com and meta-data for all goals scored (namely the current score as well as the minute of the goal) collected from the official website of the English Premier League http://premierleague.com. Moreover, short textual messages (so-called tweets) were obtained from the microblogging platform Twitter for each match covering the day of the game and including the official match hashtag (e.g. #ARSMUN for the match Arsenal vs. Manchester United) making use of the official Twitter API (Twitter API 2020). Information on the tweets includes the textual data itself as well as the exact date of creation which we relabelled to the time within the match (i.e. -30 for a tweet created 30 min prior to the match and 38.5 for a tweet created after 38.5 min of match time). A total of 3,139,441 tweets were collected, the final analysis only included tweets written one hour prior to the match or during the actual match time adding up to 1,765,379 tweets. Please note that both the Twitter Search API used within this study and the real-time Streaming API only provide a sample of the full available data.

### Feature extraction

Solely the tweets in the final analysis consist of more than 25 million words and due to the volume and the highly unstructured nature of textual data, it is not straightforward to extract machine-useable information, which underlines the importance of an elaborate feature extraction process. English tweets including the official hashtag of a match have been collected. No tweets in other languages and no additional hashtags or search terms related to the match or any official or unofficial hashtags related to the premier league and the clubs were considered. Pre-processing of tweets included removing of content other than evaluable words, like URLs, mentions, punctuation, hashtag signs, emoticons, characters and digits. Moreover, known contractions and acronyms were replaced with full forms and intentionally misspelled words (like e.g. “niiiiiiiice”) were doubled (“nice nice”) to correct for the expressed intensification. Cleaned tweets were then analysed by three different lexicon-based sentiment analysis methods, namely the commercial LIWC 2015 software (Pennebaker et al. 2015) as well as the QDAP dictionary (Rinker 2013) and the SenticNet4 lexicon based on the work of Cambria et al. (2016). Finally, an average score of positivity and negativity was assigned to each tweet. For more details, we refer to Wunderlich and Memmert (2020), who validated this exact method using football-specific Twitter data and reported a reasonable accuracy in applications with a sufficiently high number of tweets. The sentiment of a tweet is defined as the difference between the positivity and the negativity score. Sentiment has been included for analyses, but excluded as a feature in classification methods for reasons of multicollinearity and redundancy. Further non-semantic features extracted from the tweets are the average number of words (based on tweets after pre-processing), hashtags and emoticons included as well as the tweet intensity, simply referring to the number of tweets. As a further in-play feature, the total number of goals scored is considered. Pre-game features are the probability for over 2.5 goals and the probability for over 1.5 goals in the second half as obtained from the pre-game betting odds by converting decimal odds to probabilities (cf. Wunderlich and Memmert 2018). The corresponding counter-probabilities (under 2.5/1.5 goals) are not considered for reasons of redundancy. Table 1 summarizes the features used for further analysis.

**Table 1:** Features used for forecasting models throughout the paper

Feature	Source	Time
Probability over 2.5 goals	Betting market	Pre-game
Probability over 1.5 goals 2nd half	Betting market	Pre-game
Number of goals scored 1st half	Match data	In-play
Average negativity score	Twitter	In-play
Average positivity score	Twitter	In-play
Tweet intensity	Twitter	In-Play
Average number words	Twitter	In-play
Average number hashtags	Twitter	In-play
Average number emoticons	Twitter	In-play

### Normalization

Throughout our analysis, two different forms of normalization are used for the Twitter based features. Using the example of number of words in a tweet, let ${w}_{mit}$ be the number of words of each of the $n$ tweets $i=1\dots n$ in match $m$ at time $t$ (where $t$ can be a minute of play or some longer time interval such as the first half). Then we define ${w}_{mt}$ to be the average number of words in all tweets from that time interval. Let $\stackrel{\sim }{{w}_{m}}$ be the average number of words in the last hour prior to the match, then $\overline{{w }_{mt}}= {w}_{mt}$/$\stackrel{\sim }{{w}_{m}}$ is the average number of words for time interval $t$ in match $m$ normalized for pre-match data. This normalization will be denoted as *pre-match normalized* throughout the paper. Further, let $\stackrel{\sim }{{w}_{t}}$ be the average number of (pre-match normalized) words in time interval $t$ across all matches, then $\widehat{{w}_{mt}}$ = $\overline{{w }_{mt}} / \stackrel{\sim }{{w}_{t}}$ is the number of words normalized for pre-match data and additionally for time, which will simply be denoted as *time normalized* subsequently.

This definition can be used in complete analogy for the other Twitter-based features, except for the number of tweets (tweet intensity), where average values to not apply. With regard to the tweet intensity, the number of tweets per minute in a certain time interval is divided by the number of tweets per minute in the last hour before the match. A pre-match normalized tweet intensity of 2 in the first half thus means that during the first half the number of tweets per minute was twice as high as before the match. Time normalization is then performed by dividing through the number of tweets per minute across all matches. Reasons and consequences of normalizing the data in the above way as well as the results based on normalized data will be outlined in the Analysis section.

### Random forest model

Random forests are an ensemble learning method based on the idea of using a multitude of decision trees (so-called forest) going back to the work of Ho (1995). Current applications commonly refer to the method developed by Breiman (2001). Random forest methods have already been applied to forecasting in football (Schaumberger and Groll 2018) and other sports (Lessmann et al. 2010). For the present analysis, random forests were implemented in Python using the RandomForestClassifier from the package sklearn.ensemble. The following hyperparameters were tested with regard to the random forest classifier: The number of trees per forest (n_estimators ranging from 250 to 1000 in steps of 250) and the maximum depth of a tree (max_depth ranging from 1 to 8). Detailed results of the hyperparameter tuning and the effect of parameters on the results are discussed in the Analysis section.

### Cross validation

To validate the accuracy of forecasting models, *k*-fold cross validation was used, i.e. the data were split into *k* subsamples using one of the subsamples as test set and the remaining data as training set (Browne 2000). The choice of the number of subsamples $k$ is a trade-off dependent on the time required for training and the total size of the data sample available. For the analysis of small time intervals data were split into 17 subsamples resulting in training sets of 6464 time intervals and test sets of 404 time intervals each.

### Forecasting accuracy

Statistical measures of forecasting accuracy are based on the idea of quantifying the difference between the forecasted probabilities and the actual outcomes. Driven by the inconsistent use of such measures in the literature, Constantinou and Fenton (2012) have assessed this topic and proposed to use the Rank Probability Score (RPS) as an adequate measure for forecasting models in football. As the over-under market just possesses two possible outcomes, the RPS can be simplified to

$$\mathrm{RPS}= {\left({p}_{1}-{o}_{1}\right)}^{2}$$

where ${p}_{1}$ is the forecasted probability of outcome 1 and ${o}_{1}$ equals 1 if outcome 1 occurred and 0 otherwise. Due to the symmetry of binary forecasts this is equivalent to calculating RPS based on the second outcome. While being the standard approach, RPS is not undisputed and also has weaknesses which have been demonstrated by Wheatcroft (2019), who suggests to use the ignorance score instead, defined as

$${\text{IGN}} = - \log_{2} (p_{i} )$$

where ${p}_{i}$ is the forecasted probability of the actual outcome $i$. For reasons of simplicity we only report the average RPS for each model in the Results section, but we have tested results for robustness by repeating analysis with IGN and did not experience any differences with regard to the main conclusions of the paper.

### Bootstrapping

To avoid any assumptions about the theoretical distribution of the data, bootstrapping methods with 10,000 resamples were used to calculate confidence intervals and as an alternative to parametric hypotheses tests when comparing forecasting models in Tables 2 and 4 as well as in Figs. 2 and 3 with regard to sections Time dependence and Goal analysis. For an overview on bootstrapping methods and details on the calculations, we refer to Efron and Tibshirani (1994). We highlight *p*-values falling below a significance level of 5% as significant throughout the paper.

## Analysis

### Preliminary analysis: difficulty of in-play forecasting

**Fig. 1:**
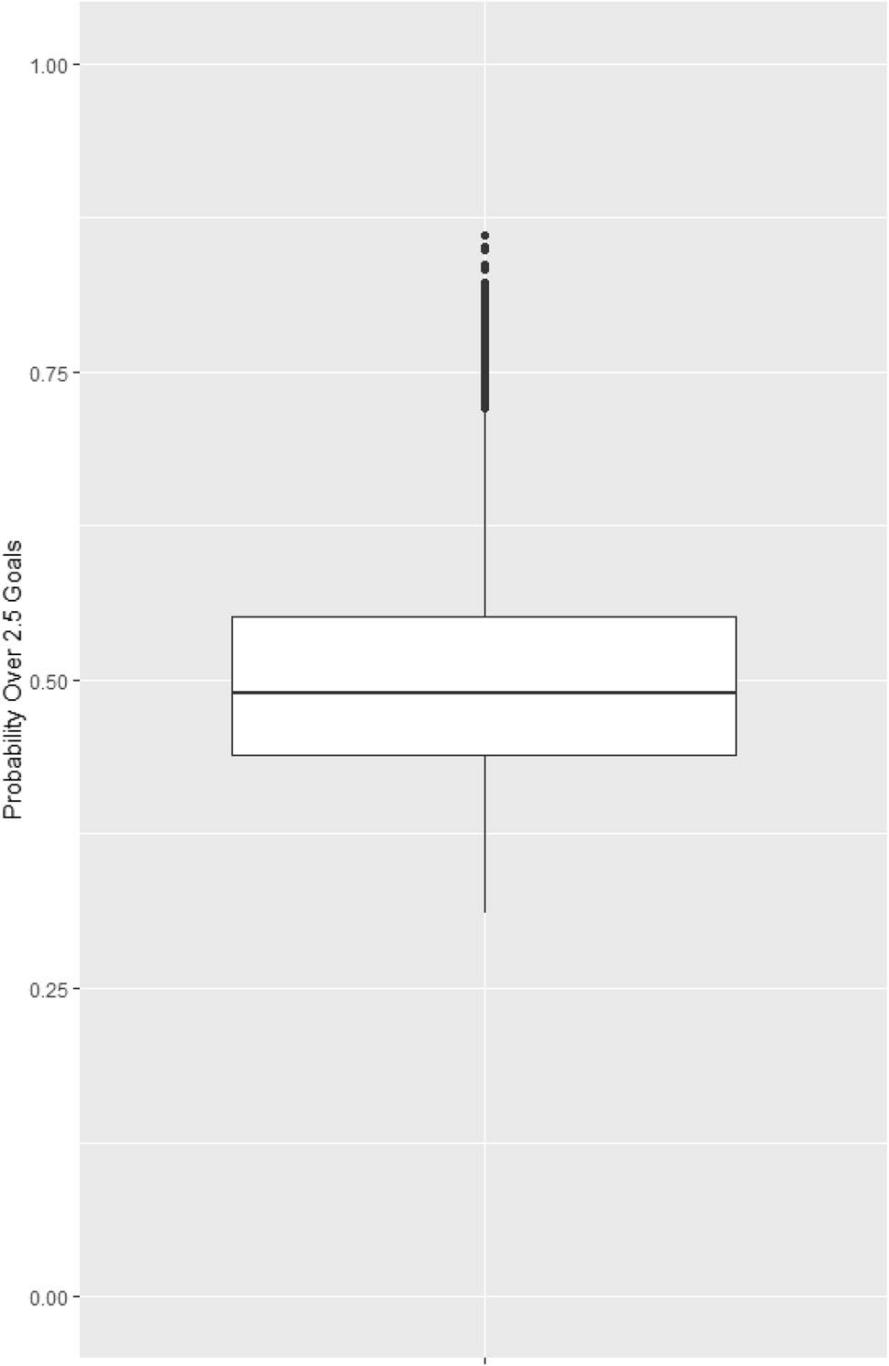
Boxplot illustrating the distribution of probabilities for over 2.5 goals across the dataset

To demonstrate the general idea of probabilistic in-play forecasting of goals and the difficulty of this task, the large dataset of more than 30,000 matches as defined in the Data section is used. The first aspect to consider is to what extent the total number of goals is predictable at all or just reflects pure random processes. Figure 1 shows a box plot of the pre-game probabilities for over 2.5 goals obtained from the betting odds. If there were no different expectations for the number of goals in a match, betting odds and thus probabilities would be constant for all matches. The dispersion of values proves that there are indeed different goal expectations, however, the expectations seem to be rather homogeneous as only one in ten matches has a lower probability than 40.3%, and only one in ten matches has a higher probability than 60.4%.

Besides the betting odds, which in principle only reflect differences in the expectations, it should also be possible to find evidence in the results directly. If matches with a systematically higher or lower total scoring intensity exist, the number of goals in the first half and second half should be correlated. Correlation was found to be *r* = 0.05 (*t*(31,910) = 13.98, *p* < 0.001), which is evidence that systematic differences in the goal expectations exist, but given the very small correlation coefficient a predominant influence of randomness exists. In summary, systematic differences in terms of expected scoring intensities across matches exist, but are highly limited.

A general predictability of the number of goals, however, does not necessarily imply that real in-play forecasting is possible at all. Thus, the next question is whether the goal expectation is predefined at the start of the match or whether information becoming available during the match helps to forecast the further course of events. In order to investigate this question, forecasts for the number of goals (i.e. probability for over 1.5 goals) in the second half are performed based on two variables: The pre-game probability for over 2.5 goals as a market reflection of goal expectation available prior to the match as well as the total number of goals actually scored in the first half as in-play information. Different numbers of goals (i.e. 2.5 goals for the complete match and 1.5 goals for the second half) were chosen to consider the option with most balanced probabilities differing due to the remaining match time. The data sample was split into 5 seasons of in-sample data (15,844 matches) and 5 seasons of out-of-sample data (16,068 matches). A total of five different forecasting models are analysed: The common naïve benchmark models *UNI* attaching a probability of 50% to over 1.5 goals for each match and *FRQ* using the observed frequency of over 1.5 goals in the in-sample data for each match (cf. Hvattum and Arntzen 2010) as well as three logistic regression models using the probability of over 1.5 goals as dependent variable and the pre-game probability for over 2.5 goals as obtained from the betting odds (*PROB*), the total number of goals scored in the first half (*GOAL*) or both variables (*BOTH*) as independent variables. Table 2 presents the average rank probability scores for each forecasting model when using the estimated model parameters to obtain forecasts for all matches in the out-of-sample dataset. In addition, pair-wise *p*-values comparing the RPS values across the models are presented.

**Table 2:** Results for various models forecasting over-under 1.5 goals in the second half

Model		RPS	UNI	FRQ	GOAL	PROB
UNI	None	0.2500	–	–	–	–
FRQ	Pre-game	0.2485	0.0069*	–	–	–
GOAL	In-play	0.2480	0.0012*	0.0467*	–	–
PROB	Pre-game	0.2434	< 0.0001*	< 0.0001*	< 0.0001*	–
BOTH	Both	0.2433	< 0.0001*	< 0.0001*	< 0.0001*	0.6073

*p-value lower than 5%

The forecasting results paint a clear picture and suggest that in-play information does have some very weak predictive value when comparing to simple benchmarks, but no additional value when controlling for pre-game information. The model *PROB* using solely the pre-game expectation significantly outperforms both benchmarks and the model *GOAL* using only in-play information. Once the pre-game information is included, the average rank probability scores for the model hardly improves if adding in-play information on the number of goals. Despite the large database the model *BOTH* using both pre-game and in-play information fails to significantly outperform *PROB*. The above results are clear evidence for the difficulty of forecasting the total number of goals in-play, yet it is not clear whether the small benefit of in-play information is based on the fact that the goal expectation is predefined prior to the match or that prior goals are just not a useful in-play predictor. For that reason, Twitter data as a potential source of in-play information are analysed in the next section. Before turning to forecasts from Twitter data, the data are analysed with respect to several other aspects, namely factors influencing tweet intensity, and the effect of time and goals on Twitter communication.

### Twitter analysis

#### Match-based analysis of tweet intensity

A first qualitative observation in the Twitter data is that differences across matches seem to only partly depend on in-play events as even before the start massive differences can occur. As an extreme example the tweet intensity for the match Manchester vs. Arsenal was more than 100 times higher than for the match Brighton vs. Burnley both pre-match and in-play. For this reason, a closer look on the reasons for varying tweet intensities in the matches shall be given. In particular, four factors potentially having influence on tweet intensity will be analysed:

##### Popularity

First, we use the average number of spectators at home matches of a team as an estimation of the general popularity of this team. The numbers were obtained from https://www.weltfussball.de and as the dataset contains matches from 18/19 to 19/20 season, the spectator numbers were averaged across both seasons. Moreover, spectators were normalized by the maximum number of spectators of any team, which yields popularities ranging from 1.0 for Manchester United with the highest number of spectators to 0.14 for Bournemouth being the least popular team. The number of spectators arguably is not a perfect representation of popularity given that the capacity of a stadium can be a highly confounding factor. Still, it can be assumed to be an easily available and transparent measure with a reasonably high correlation to popularity. To account for both teams’ popularities in a match concurrently, the popularity of a match is determined by multiplying the popularity of both teams.

##### Goals

Match events in general and goals in particular can be assumed to stimulate Twitter activity. Thus, the total number of goals is used as the second factor potentially influencing the tweet intensity.

##### Scoreline

The scoreline during a match determines whether the game has already been decided or whether the results is still open. This may take influence on the behaviour of Twitter users in several ways. It could be argued that a close scoreline captivates the audience and stimulates tweet intensity. At the same time, matches being decided early may stimulate early analysis of results including joy about an upcoming victory or discussing reasons for a lost match. We summarize the scoreline of a match by summing up the length of time intervals in a match where both teams differ at least by two goals and consequently a single goal would not significantly alter the match outcome. For example, a match ending 2–0 with the second goal being scored after exactly 60 min has been “decided” for 30 min.

##### Weekend

Finally, external factors neither related to the teams, nor related to the events in a match may have an influence on the possibility and motivation for fans to watch and tweet on football matches. Thus, we introduce a dummy variable indicating whether a match took place on a weekend (Saturday or Sunday) or during the week (Monday–Friday).

Three linear regression models were fitted using the tweet intensity (pre-game, in-game, and total, respectively) as dependent variable and the four factors as independent variables. All three regression models indicate a significant influence of the factors on the tweet intensities: *F*(4,399) = 91.72, *p* < 0.001, ${{R}^{2}}_{\mathrm{adj}}=0.474$ for pre-game tweet intensity; *F*(4,399) = 88.62, *p* < 0.001, ${{R}^{2}}_{\mathrm{adj}}=0.465$ for in-game tweet intensity; *F*(4,399) = 92.54, *p* < 0.001, ${{R}^{2}}_{\mathrm{adj}}=0.476$ for total tweet intensity. The detailed results for each factor are summarized in Table 3.

**Table 3:** Results for the linear regression model using tweet intensities as dependent variable on a match level. Separate models are summarized for tweet intensity pre game, tweet intensity in game and total tweet intensity

Variable	IntensityPreGame					IntensityInGame					Intensity				
	Coefficient	Standard error	beta	*t*	*p*-value	Coefficient	Standard error	beta	*t*	*p*-value	Coefficient	Standard error	beta	t	p**-value**
Popularity	3324.44	174.95	0.69	19.00	< 0.001*	20,389.08	1116.81	0.67	18.26	< 0.001*	23,713.52	1263.47	0.68	18.77	< 0.001*
Goals	− 3.56	20.11	− 0.01	− 0.18	0.86	363.58	128.35	0.12	2.83	< 0.01*	360.02	145.20	0.10	2.48	0.014*
Scoreline	1.11	1.40	0.03	0.79	0.43	14.88	8.97	0.07	1.66	0.10	15.99	10.15	0.06	1.56	0.12
Weekend	− 59.89	66.80	− 0.03	− 0.90	0.37	162.45	426.42	0.01	0.38	0.70	102.56	482.42	0.01	0.21	0.83
Constant	− 238.93	90–94		− 2.63	< 0.01*	− 2845.20	580.52		− 4.90	< 0.001*	− 3084.13	656.75		−4.70	< 0.001*

*Significant at 5% level

Results are evidence that tweet intensity both pre-game and in-game is highly significantly (*p* < 0.001) influenced by popularity, while there is no significant influence of the weekend on the number of tweets. Naturally, Goals and Scoreline being linked to match events unknown pre-game do not possess any significant influence on pre-game intensities. In-game, however, the number of goals significantly increases (*p* < 0.01) the number of tweets, indicating an increased stimulation of tweets via goals that will be analysed in more detail in the section Goal analysis. Scoreline does not have a significant influence on in-game tweet intensity (*p* = 0.10), however, there is a slight tendency of more tweets in case of already decided matches. Driven by the larger number of in-game tweets, the results for total tweet intensity are largely consistent with the results for in-game tweet intensity.

Given the heterogeneity of matches in terms of large differences in tweet intensity and the fact that other features (yet to a lower degree) differ pre-match, it seems unreasonable to draw any conclusion about in-match processes from non-normalized values. For this reason, the subsequent analysis is based on pre-match normalized data as explained in the Method section.

#### Time dependence

**Fig. 2:**
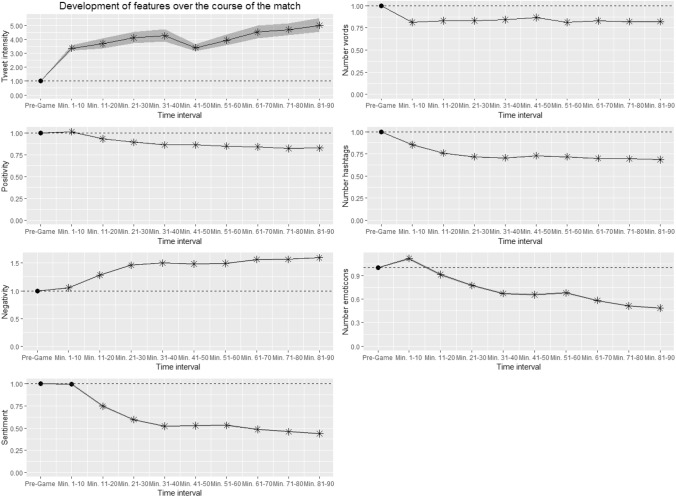
Evolvement of features over the course of the match. Asterisks indicate a *p*-value of lower than 0.05 when comparing the respective time interval to pre-game

Before considering potential predictive value, it seems reasonable to take a more general look at what happens over the course of the match and directly before and after goals. Figure 2 illustrates the evolvement of features over time during the matches for ten time intervals (the hour pre-game and nine intervals of 10 min each within the match, as well as 95% confidence intervals. Please note that pre-game values equal 1.0 for each feature due to the pre-match normalization.

The tweet intensity jumps when the match starts and slightly increases over the course of the match. Interestingly, the overall sentiment of tweets decreases due to decreasing positivity and increasing negativity. Tweets get shorter once the match starts as the number of words drops after the kick-off. The number of hashtags and emoticons decreases as well, which can only partly be attributed to the shorter tweets. Confidence intervals are hardly visible in the figure except for the tweet intensity, where the sample size is about 400 matches compared to almost 2 million tweets for the other features. The narrow confidence intervals (even for the tweet intensity) suggest that all results are highly robust. Some interesting conclusions can be drawn from the evolvement of features: First, if analysing the time intervals before and after goals a useful normalization for time is needed. Therefore, the time normalized data, as described in the Method section, is used for all further analyses. Second, football fans seem to be the happiest before the kick-off and a football match does not seem to be good for the mood (at least of tweeters). In summary, one can say that anticipation clearly is the most beautiful kind of joy.

#### Goal analysis

**Fig. 3:**
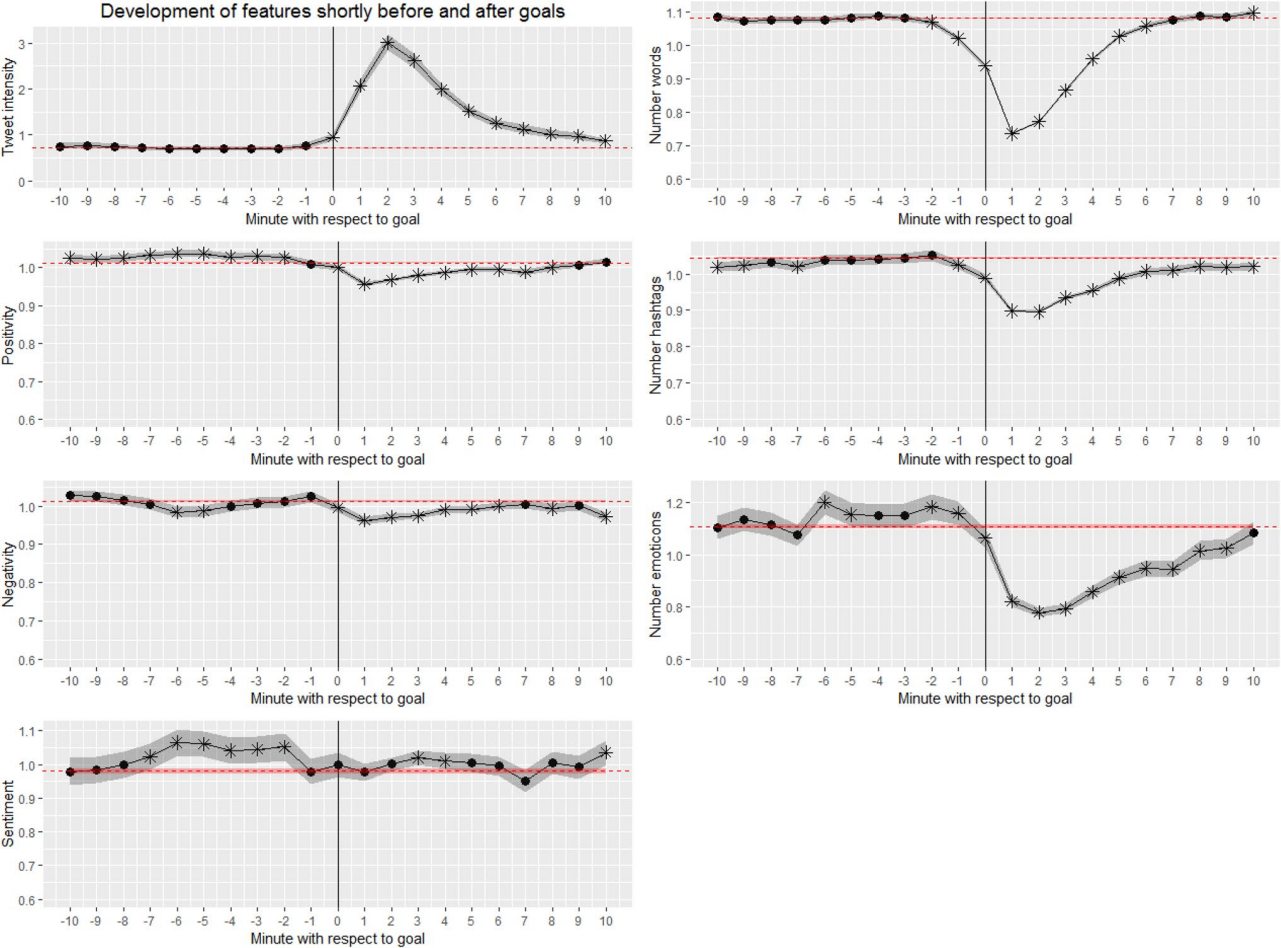
Evolvement of features shortly before and after goals. The vertical line illustrates the time when the goal was scored. The horizontal line refers to the benchmark of tweets that were neither written shortly before nor after a goal. Asterisks indicate a *p*-value of lower than 0.05 when comparing the respective minute to the benchmark

Usage of the time normalized data makes it possible to take a direct look at what happens before and after goals are scored. Therefore, a minute value with respect to goals was assigned to each tweet. Negative values were attached to tweets that were posted within the last 10 min before a goal (e.g. -7 if the tweet was posted 7 min before the goal). Analogously, positive values were attached to tweets posted within the 10 min following a goal and 0 if the tweet was posted in the same minute of the goal. Tweets that were posted before or after several different goals cannot be unambiguously assigned and thus were excluded from analysis. Tweets that are not close in time to any goal were put into an additional category and used as a benchmark. Figure 3 illustrates what happens to the various Twitter features in the 10 min before and after a goal. The dotted vertical line refers to the benchmark of tweets that are independent from goals, and the grey areas refer to 95% confidence intervals. The analysis includes a total of 1118 goals scored in the matches from our dataset.

Again, confidence intervals are narrow or even hardly visible indicating the robustness of results. A clear and intuitive interpretation can be given for the time interval after the goals: Goals evoke a large number of relatively short tweets. While the number of tweets increases by a factor of three shortly after the goal, the tweet length decreases by roughly 30%. The effects for hashtags and emoticons are partly attributable to the shortness of the tweets. Only small effects are visible with regard to the sentiment analysis, where surprisingly both negativity and positivity slightly decreases resulting in a pretty stable overall tweet sentiment. Please note that the tweets are attached to a match and not to particular teams, which means that we cannot distinguish between the fans of the scoring and the conceding team. In the forecasting context, the idea is to find signs that are already present in the data during the minutes before the goals are scored. Most features are in line with the benchmark and thus do not support this idea. However, a slightly increased positivity and overall sentiment can be found prior to the goals, potentially being a weak early indication of goals. The same is true for emoticons, however, being less clear.

#### Analysis of words and topics

**Fig. 4:**
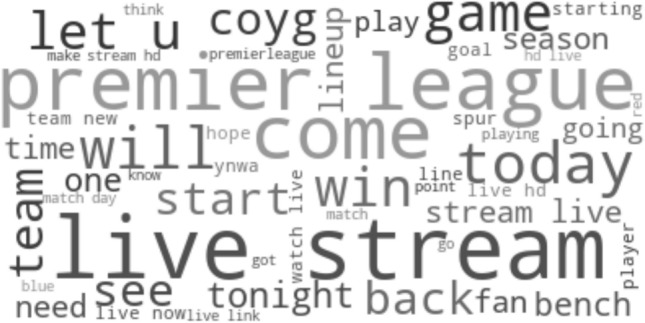
Wordcloud visualizing frequently used words from tweets written pre-match, i.e. within the last hour before the match

**Fig. 5:**
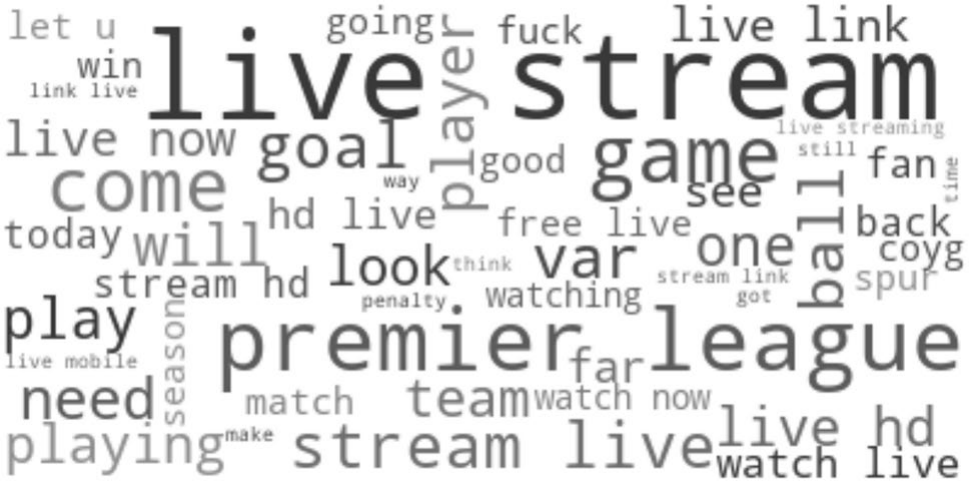
Wordcloud visualizing frequently used words from tweets written during the first half of a match, but excluding tweets being written shortly before and after goals

**Fig. 6:**
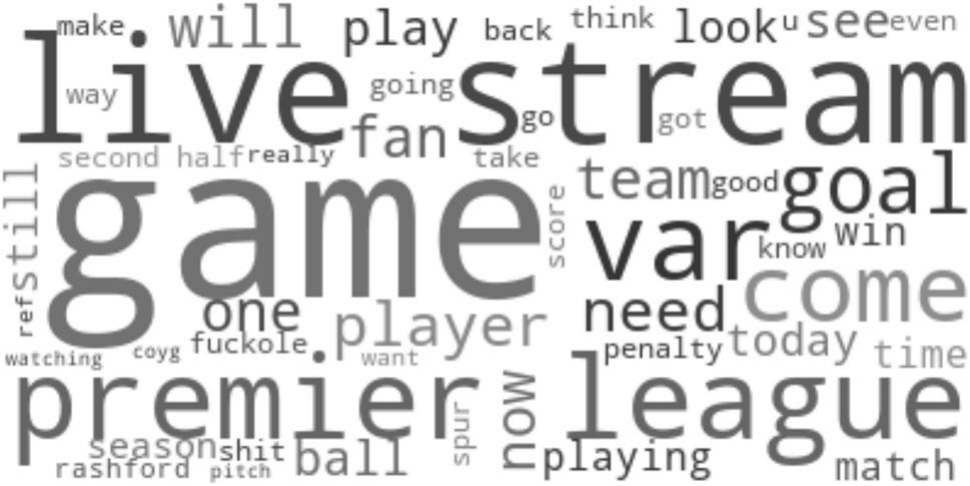
Wordcloud visualizing frequently used words from tweets written during the second half of a match, but excluding tweets written shortly before and after goals

**Fig. 7:**
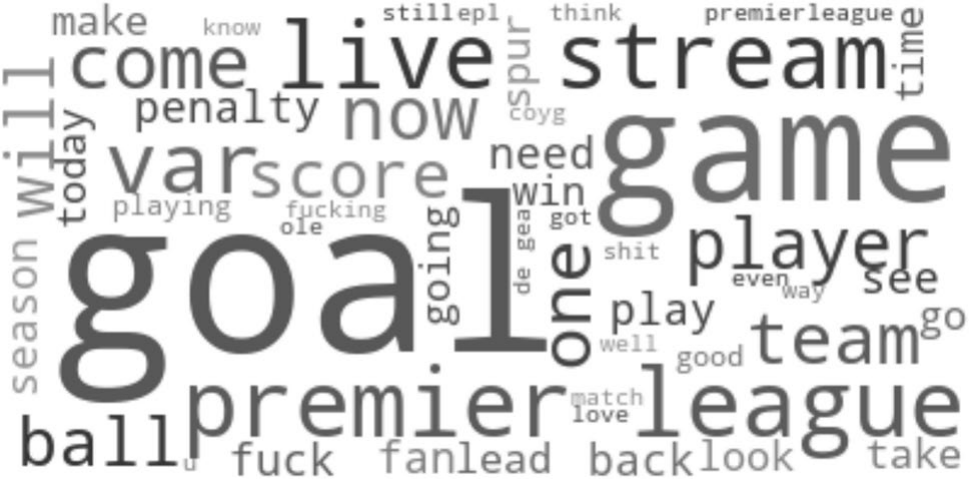
Wordcloud visualizing frequently used words from tweets written shortly after goals

The analyses so far have taken account of the number of words or the sentiment of words, but not visualized the communication in a more detailed way. In order to gain insights on the topics and frequently used words in association with a match, four different wordclouds representing different phases before and during the matches are used. Figure 4 analyses pre-match communication and thus refers solely to tweets written in the last hour before a match started. Figures 5 and 6 refer solely to tweets written during the first half and the second half, respectively. However, in order to capture general instead of event-based communication on the matches, those tweets being associated to one or more goals (i.e. written in the 10 min before or after goals) were not considered. Finally, Fig. 7 analyses event-based communication and thus includes tweets written in the 10 min following a goal. Tweets that cannot unambiguously associated with a single goal have not been considered in consistency with the section Goal analysis. Wordclouds were created by means of the python package *wordcloud* (version 1.8.1) using 50 as the maximum number of words. The official hashtags of the matches, all team names and known acronyms of team names (such as “lfc” for “liverpool football club”) were not considered, as well as the predefined list of typical stopwords including words like e.g. “it”, “was” or “this”.

Pre-game communication includes plenty of words related to broadcasting of the match, such as “*live stream*”, “watch live” and “hd”. Moreover, several words including “start”, “starting”, “today”, “tonight” or “now” directly refer to the upcoming start of the match. Finally, there seems to be noticeable discussion on which players were chosen to play or not play by the coach, evidenced by words like “lineup”, “bench”, “player” and possibly also “team” or “starting”. Please note that unusual terms like “coyg” (come on you gunners) or “ynwa” (you’ll never walk alone) refer to football-related acronyms that were not contained in our list of known acronyms and thus remained included in the data.

Communication during the match is still subject to a lot of discussion on how to follow broadcasts of the match, as words like “live stream”, “live” and “hd” stay highly present. The presence of words like “play”, “player”, “playing” or “fan” can be considered very general and expectable for communication during the match, that is not associated to goals. In general, differences between first and second half seem to be rather limited, except for the prominent role of the word “game” in the second half. This suggests that towards the end of the match or given a clear scoreline, users tend to already discuss the game as a whole, summarize it or draw conclusions from it.

Communication directly following a goal is naturally strongly influenced by words in direct association to the goal (“goal”, “score”, “lead”), or related to discussing the circumstances of a goal, such as “var” (video assistant referee) or “penalty”. The large occurrence of the word “game” might again indicate that once a goal decides a match, it is already discussed as a whole. Moreover, some expectable expressions of emotional reactions like “good” or “shit” are included, however, by far not dominating the wordcloud.

#### In-play goal forecast

In order to answer the question whether the information in the data is sufficient to forecast goals in-play, small time intervals were considered. Matches were split into intervals of 5 min and normalized Twitter features were calculated accordingly. The variable to be forecasted is an indicator of whether a goal was scored in the next time interval and the last interval of 5 min per match was consequently excluded from the data. This results in a sample of 6868 time intervals. No betting odds are available for time intervals of 5 min, therefore* ODDS* refers to a logistic regression based on the betting odds of over-under 2.5 goals as well as over-under 1.5 goals in the second half.

**Fig. 8:**
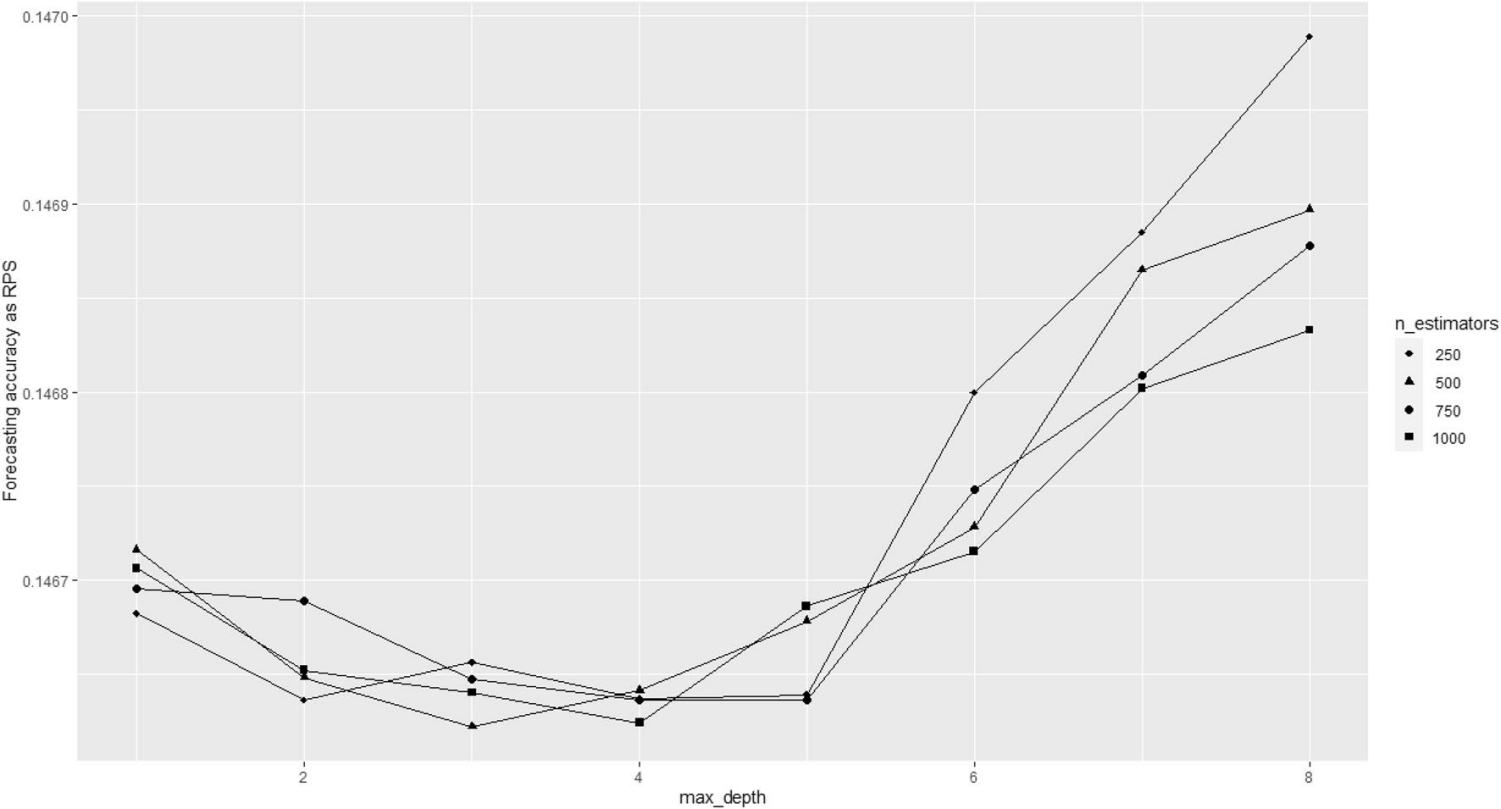
Results of hyperparameter tuning for the random forest model. The forecasting accuracy as RPS is illustrated in dependence of the number of trees (n_estimators) and the maximum tree depth (max_depth)

Results show that *UNI* is an unreasonable choice for the short time intervals, which is attributable to the fact that goal scoring probabilities for intervals of 5 min are way smaller than 50%. As expectable, *FRQ* representing the lowest level of information also possesses the weakest predictive accuracy. *ODDS* possesses the highest predictive quality, which is in line with the notion that betting odds are a strong predictor of football matches. The main result is that *LR* and *RF*, although including additional in-play information both fail to outperform pre-game information based on the betting odds. As such, Twitter data did not improve pre-game forecasts for the number of goals in matches. Except for *UNI*, all other models are pretty close in terms of accuracy with the only significant difference between *FRQ* and *ODDS*. This underlines that in-play forecasting seems to be a difficult task. Results for the hyperparameter tuning are summarized in Fig. 8, which shows that increasing the number of trees had a very limited effect, while the optimal maximum depth lies around 3 to 4. The results in Table 4 refer to the optimal specification of n_estimators = 500 and max_depth = 3. Please note that the hyperparameters did not have major effects on the forecasting accuracy of *RF* ranging from 0.1466 to 0.1470. More importantly, for none of the hyperparameters tested, a significant difference between *RF* and *LR* or *ODDS* was found. As such, the hyperparameter selection does not affect any of the results of the present study.

**Table 4:** Results for forecasting goals in time intervals of 5 min from the preceding time interval

Model	Information	RPS	*p*-value compared to			
			UNI	FRQ	LR	RF
UNI	None	0.2500	–	–	–	–
FRQ	Pre-game	0.1469	< 0.0001*	–	–	–
LR	Both	0.1467	< 0.0001*	0.4362	–	–
RF	Both	0.1466	< 0.0001*	0.1578	0.7966	–
ODDS	Pre-game	0.1465	< 0.0001*	0.0452*	0.3642	0.3584

*p-value lower than 5%

## Discussion

The results of the present study shed light on three different aspects of in-play forecasting with Twitter data, namely in-play forecasting in general, a detailed analysis of Twitter communication over the course of matches and the value of Twitter in in-play forecasting in football.

The preliminary analysis suggests that in-play forecasting of goals in general is a difficult task. Results are evidence for the limited value of in-play information (i.e. goals) to forecast the further course of a match when compared to betting odds as pre-game information, a fact that football players, coaches, match analysts, broadcasters and fans would probably strongly deny. Possible explanations are the high predictive quality of betting odds in football forecasting (Forrest et al. 2005; Hvattum and Arntzen 2010; Štrumbelj and Šikonja 2010) and the significant role of randomness in goal scoring in football (Brechot and Flepp 2020; Lames 2018, Wunderlich et al. 2021). Moreover, the result is in line with Wunderlich and Memmert (2018) who showed that betting odds of prior matches possess more predictive value than the results of the matches themselves.

The analysis of tweet intensity revealed that both in-play and pre-game, tweet intensity is predominantly driven by the popularity of the two teams competing. Moreover, tweet intensity is increased in-play in matches with a higher number of total goals scored. The analysis of time dependence and goal analysis reveal how the reactions of Twitter users change over the course of matches and after goals are scored. Before the matches start less, but longer tweets are written, when compared to during the match, which is explainable by a heightened interest and faster sequence of events in-play. In terms of the topics and words contained, pre-game tweets are highly influenced by communication on how to follow broadcasts of the match and discussing which players are playing. Differences between communication in the first and second half are highly limited, while tweets directly following goals are naturally dominated by discussion on the score, the goal itself and its possible causes. The most striking result with regard to time dependence is a steadily increasing negativity and a steadily decreasing positivity while the match evolves, resulting in a clearly decreasing sentiment. It seems that fans (or at least those active on Twitter) tend to be disappointed by football matches, possibly caused by unjustified high expectations before and at the beginning of matches. The use of Twitter data and sentiment analysis techniques enables researchers to investigate perception and psychological reactions of users during football matches. Further research with a psychological focus could investigate which mechanisms drive the disappointment of fans during matches.

The analysis of minutes before and after goals reveals the reaction to goals, in particular a dramatic increase in tweet intensity where tweets are significantly shorter and a resulting lower number of hashtags and emoticons. The most unintuitive and difficult to explain result is the slightly lower negativity and positivity directly after goals. It is important to note that the tweets in our database are assigned to the match and not to a single team, thus emotions of both teams’ fans should be included which makes an unchanged overall sentiment comprehensible. However, even if including fans of the team scoring, the team receiving and even neutral observers, one would at least expect an increased emotionality as a reaction to the goal. One explanation could be neutral tweets that have a descriptive and no evaluative expression (e.g. “Penalty for The Red Devils. Rashford steps up and CONVERTS! Manchester United 1–0 Chelsea.”) or tweets that were potentially written with a lot of emotion, but do not include any words with a clear positive or negative connotation identifiable by a sentiment analysis algorithm (e.g. "GOOO[…]OOOL!!!! Rashford!!! 1–0 United!!!). With regard to the sentiments, although being validated in football, textual data are highly domain-specific and increased accuracy might be achievable if using domain-specific methods such as football-specific lexica of words. A more detailed analysis on what drives this unintuitive result, however, is beyond the scope of this study.

While the Twitter data clearly react to goals scored, a main focus of our approach was to test Twitter data for possible predictive value. The present data clearly do not support the idea that in-play Twitter data have predictive value as forecasts based on pre-game betting odds were not outperformed by a logistic regression model as well as a random forest model including in-play Twitter information. The fact that random forest models did not outperform logistic regression and hyperparameter tuning did have very limited effects on the accuracy suggests that this is actually attributable to the missing informative value of the Twitter data and not to the selection of methods. Put simply, we could not extract information from Twitter data that helps to forecast upcoming goals. Three possible aspects could explain this result. First, the in-play predictability seems to be very limited in general as previously demonstrated. Further studies investigating in-play notational data or positional data could shed more light on the question to which degree in-play forecasting is possible at all. Second, Twitter data might not include information that is relevant for forecasting. In a way, this is surprising as Twitter can be seen as a source of crowd wisdom and such sources have been shown to be highly valuable in forecasting football (Forrest et al. 2005; Peeters 2018; Spann and Skiera 2009). On the other side, Twitter is not a vehicle directly related to forecasting such as the betting market or prediction markets and moreover information is not easily extractable from Twitter. Thus, the third possible aspect is that the information reflected in Twitter data might not have been extracted effectively. Textual data are highly unstructured which makes the extraction of information difficult and leads to a limited degree of accuracy for sentiment analysis techniques (Wunderlich and Memmert 2020). Further progress in this domain can be expected as sentiment analysis is a highly relevant topic in computer science (Mäntylä et al. 2018; Piryani et al. 2017), nevertheless it will remain challenging to algorithmically reproduce human understanding of textual data. The problem of extracting relevant data might be aggravated by the short time intervals of 5 min yielding limited tweet samples and a higher randomness in the features. To account for the issue of short time intervals, we repeated analysis using data from the complete first half of a match to forecast the number of goals in the second half of a match. Despite larger time intervals, results implied the same conclusions, which suggest that the limited in-play predictive value is not attributable to the small time intervals.

In experimental research, the present results could be assessed as a null result as they do not support the notion of predictive in-play value of Twitter data and question the general value of in-play information including goals. Still, this is surprising and valuable information to coaches, match analysts and broadcasters who should question carefully to what extent in-play information can be used at all to draw conclusions on the further course of a match.

## Conclusions

The present approach investigates in-play forecasting of football matches in general and a Big Data approach using Twitter data in particular. Results are evidence that in-play forecasting of goals is a highly challenging task as information gathered in-play (both basic events like goals and textual data from Twitter) are not improving forecasting accuracy when compared to pre-game information. In addition, results suggest that the fans’ perception of a match gets more and more negative over time as the sentiment of tweets on Twitter is decreasing over the course of the match.

## Declarations

### Data availability

No data with regard to the direct content of tweets will be published or forwarded in line with the Developer Agreement and Policy of the Twitter API. All further data used within this study are available in the public domain and the respective sources have been mentioned in the manuscript.

## References

[CR1] Akhtar S, Scarf P (2012). Forecasting test cricket match outcomes in play. Int J Forecast.

[CR2] Andersson P, Edman J, Ekman M (2005). Predicting the World Cup 2002 in soccer: performance and confidence of experts and non-experts. Int J Forecast.

[CR3] Asif M, McHale IG (2016). In-play forecasting of win probability in one-day international cricket: a dynamic logistic regression model. Int J Forecast.

[CR4] Berrar D, Lopes P, Davis J, Dubitzky W (2019). Guest editorial: special issue on machine learning for soccer. Mach Learn.

[CR5] Bollen J, Mao H, Zeng X-J (2011). Twitter mood predicts the stock market. J Comput Sci.

[CR6] Boshnakov G, Kharrat T, McHale IG (2017). A bivariate Weibull count model for forecasting association football scores. Int J Forecast.

[CR7] Brechot M, Flepp R (2020). Dealing with randomness in match outcomes: how to rethink performance evaluation in European club football using expected goals. J Sports Econ.

[CR8] Breiman L (2001). Random forests. Mach Learn.

[CR9] Brown A, Rambaccussing D, Reade JJ, Rossi G (2017). Forecasting with social media: evidence from tweets on soccer matches. Econ Inq.

[CR10] Browne (2000). Cross-validation methods. J Math Psychol.

[CR11] Cambria E, Poria S, Bajpai R, Schuller B (2016) SenticNet 4: a semantic resource for sentiment analysis based on conceptual primitives. In: Proceedings of COLING 2016, the 26th international conference on computational linguistics: technical papers, pp 2666–2677

[CR12] Constantinou AC, Fenton NE (2012). Solving the problem of inadequate scoring rules for assessing probabilistic football forecast models. J Quant Anal Sports.

[CR13] Dick U, Brefeld U (2019). Learning to rate player positioning in soccer. Big Data.

[CR14] Dixon MJ, Robinson ME (1998). A birth process model for association football matches. Statistician.

[CR15] Easton S, Uylangco K (2010). Forecasting outcomes in tennis matches using within-match betting markets. Int J Forecast.

[CR16] Efron B, Tibshirani RJ (1994). An introduction to the bootstrap.

[CR17] Fan M, Billings A, Zhu X, Yu P (2020). Twitter-based BIRGing: big data analysis of english national team fans during the 2018 FIFA world cup. Commun Sport.

[CR18] Forrest D, Goddard J, Simmons R (2005). Odds-setters as forecasters: the case of English football. Int J Forecast.

[CR19] Gayo-Avello D (2013). A meta-analysis of state-of-the-art electoral prediction from Twitter data. Soc Sci Comput Rev.

[CR20] Goddard J, Asimakopoulos I (2004). Forecasting football results and the efficiency of fixed-odds betting. J Forecast.

[CR21] Godin F, Zuallaert J, Vandersmissen B, de Neve W, van de Walle R (2014) Beating the bookmakers: leveraging statistics and Twitter microposts for predicting soccer results. In: KDD Workshop on large-scale sports analytics

[CR22] Grunz A, Memmert D, Perl J (2012). Tactical pattern recognition in soccer games by means of special self-organizing maps. Hum Mov Sci.

[CR23] Heuer A, Rubner O (2012). How does the past of a soccer match influence its future? Concepts and statistical analysis. PLoS ONE.

[CR24] Ho TK (1995) Random decision forests. In: Proceedings of the third international conference on document analysis and recognition, August 14–16, 1995, Montréal, Canada. IEEE Computer Society Press, Los Alamitos, pp 278–282. 10.1109/ICDAR.1995.598994

[CR25] Hubáček O, Šourek G, Železný F (2019). Exploiting sports-betting market using machine learning. Int J Forecast.

[CR26] Huberty M (2015). Can we vote with our tweet? On the perennial difficulty of election forecasting with social media. Int J Forecast.

[CR27] Hvattum LM, Arntzen H (2010). Using ELO ratings for match result prediction in association football. Int J Forecast.

[CR28] Jungherr A, Jürgens P, Schoen H (2011) Why the pirate party won the German election of 2009 or the trouble with predictions: a response to Tumasjan, A., Sprenger, T. O., Sander, P. G., & Welpe, I. M. “Predicting Elections With Twitter: What 140 Characters Reveal About Political Sentiment”. Soc Sci Comput Rev 30(2):229–234. 10.1177/0894439311404119

[CR29] Karlis D, Ntzoufras I (2003). Analysis of sports data by using bivariate Poisson models. J R Stat Soc Ser D (The Stat).

[CR30] Killick EA, Griffiths MD (2019). In-play sports betting: a scoping study. Int J Ment Heal Addict.

[CR31] Kolbinger O, Knopp M (2020). Video kills the sentiment-exploring fans' reception of the video assistant referee in the English premier league using Twitter data. PLoS ONE.

[CR32] Koopman SJ, Lit R (2015). A dynamic bivariate Poisson model for analysing and forecasting match results in the English premier league. J R Stat Soc A Stat Soc.

[CR33] Koopman SJ, Lit R (2019). Forecasting football match results in national league competitions using score-driven time series models. Int J Forecast.

[CR34] Kovalchik S, Reid M (2019). A calibration method with dynamic updates for within-match forecasting of wins in tennis. Int J Forecast.

[CR35] Lames M (2018). Chance involvement in goal scoring in football—an empirical approach. Ger J Exerc Sport Res.

[CR36] Lasek J, Szlávik Z, Bhulai S (2013). The predictive power of ranking systems in association football. Int J Appl Pattern Recognit.

[CR37] Lessmann S, Sung M-C, Johnson JE (2010). Alternative methods of predicting competitive events: an application in horserace betting markets. Int J Forecast.

[CR38] Lopez-Gonzalez H, Griffiths MD (2016). Is European online gambling regulation adequately addressing in-play betting advertising?. Gaming Law Rev Econ.

[CR39] Maher MJ (1982). Modelling association football scores. Stat Neerl.

[CR40] Mäntylä MV, Graziotin D, Kuutila M (2018). The evolution of sentiment analysis—a review of research topics, venues, and top cited papers. Comput Sci Rev.

[CR41] Memmert D, Raabe D (2018) Data analytics in football. Routledge, Abingdon. 10.4324/9781351210164

[CR42] Peeters T (2018). Testing the wisdom of crowds in the field: transfermarkt valuations and international soccer results. Int J Forecast.

[CR43] Pennebaker JW, Boyd RL, Jordan K, Blackburn K (2015) The development and psychometric properties of LIWC2015. University of Texas at Austin. 10.15781/T29G6Z

[CR44] Piryani R, Madhavi D, Singh VK (2017). Analytical mapping of opinion mining and sentiment analysis research during 2000–2015. Inf Process Manag.

[CR45] Rein R, Memmert D (2016). Big data and tactical analysis in elite soccer: future challenges and opportunities for sports science. Springerplus.

[CR46] Rinker TW (2013) qdapDictionaries: dictionaries to accompany the qdap package. Retrieved from http://github.com/trinker/qdapDictionaries

[CR47] Schaumberger G, Groll A (2018). Predicting matches in international football tournaments with random forests. Stat Model.

[CR48] Schumaker RP, Jarmoszko AT, Labedz CS (2016). Predicting wins and spread in the premier league using a sentiment analysis of twitter. Decis Support Syst.

[CR49] Spann M, Skiera B (2009). Sports forecasting: a comparison of the forecast accuracy of prediction markets, betting odds and tipsters. J Forecast.

[CR50] Štrumbelj E, Šikonja MR (2010). Online bookmakers’ odds as forecasts: the case of European soccer leagues. Int J Forecast.

[CR51] Tumasjan A, Sprenger TO, Sandner PG, Welpe IM (2010). Predicting elections with Twitter: what 140 characters reveal about political sentiment. Icwsm.

[CR52] Twitter API (2020) Retrieved from https://developer.twitter.com/

[CR53] Wheatcroft E (2019) Evaluating probabilistic forecasts of football matches: the case against the ranked probability score. https://arxiv.org/abs/1908.08980

[CR54] Wheatcroft E (2020). A profitable model for predicting the over/under market in football. Int J Forecast.

[CR55] Wunderlich F, Memmert D (2018). The betting odds rating system: using soccer forecasts to forecast soccer. PLoS ONE.

[CR56] Wunderlich F, Memmert D (2020). Innovative approaches in sports science—lexicon-based sentiment analysis as a tool to analyze sports-related Twitter communication. Appl Sci.

[CR65] Wunderlich F, Seck A, Memmert D (2021). The influence of randomness on goals in football decreases over time. An empirical analysis of randomness involved in goal scoring in the English Premier League. J Sports Sci.

[CR57] Yu Y, Wang X (2015). World cup 2014 in the Twitter world: a big data analysis of sentiments in U.S. sports fans’ tweets. Comput Hum Behav.

[CR58] Zhang X, Fuehres H, Gloor PA (2011). Predicting stock market indicators through Twitter “I hope it is not as bad as I fear”. Proc Soc Behav Sci.

[CR59] Zou Q, Song K, Shi J (2020). A Bayesian in-play prediction model for association football outcomes. Appl Sci.

